# Leopard density and the ecological and anthropogenic factors influencing density in a mixed-use landscape in the Western Cape, South Africa

**DOI:** 10.1371/journal.pone.0293445

**Published:** 2023-10-27

**Authors:** Kyle Hinde, Anita Wilkinson, Silindokuhle Tokota, Rajan Amin, M. Justin O’Riain, Kathryn S. Williams

**Affiliations:** 1 Institute for Communities and Wildlife in Africa (iCWild), University of Cape Town, Cape Town, South Africa; 2 The Cape Leopard Trust, Cape Town, South Africa; 3 Conservation Programmes, Zoological Society of London, London, United Kingdom; 4 Department of Anthropology, Durham University, Durham, United Kingdom; Smithsonian Conservation Biology Institute, UNITED STATES

## Abstract

Large carnivores face numerous threats, including habitat loss and fragmentation, direct killing, and prey depletion, leading to significant global range and population declines. Despite such threats, leopards (*Panthera pardus*) persist outside protected areas throughout most of their range, occupying diverse habitat types and land uses, including peri-urban and rural areas. Understanding of leopard population dynamics in mixed-use landscapes is limited, especially in South Africa, where the majority of leopard research has focused on protected areas. We use spatially explicit capture-recapture models to estimate leopard density across a mixed-use landscape of protected areas, farmland, and urban areas in the Overberg region of the Western Cape, South Africa. Data from 86 paired camera stations provided 221 independent captures of 25 leopards at 50 camera trap stations with a population density estimate of 0.64 leopards per 100 km^2^ (95% CI: 0.55–0.73). Elevation, terrain ruggedness, and vegetation productivity were important drivers of leopard density in the landscape, being highest on elevated remnants of natural land outside of protected areas. These results are similar to previous research findings in other parts of the Western Cape, where high-lying natural vegetation was shown to serve as both a refuge and a corridor for leopard movement in otherwise transformed landscapes. Given the low leopard density and the prevalence of transformed land intermixed with patches of more suitable leopard habitat, prioritising and preserving connectivity for leopards is vital in this shared landscape. Ecological corridors should be developed in partnership with private landowners through an inclusive and multifaceted conservation strategy which also incorporates monitoring of and rapid mitigation of emerging threats to leopards.

## Introduction

Housing and food requirements to support the global human population of over 8 billion people place extreme pressure on land and natural resources. Over half of the planet’s land is shared by both wildlife and people [1]. Large carnivores with their broad spatial requirements, long generation time, and high trophic level are extremely vulnerable to habitat loss and land transformation [2, 3]. Living in shared agricultural areas presents challenges for both carnivores and people. Negative effects for wildlife and humans are common at this interface, with wildlife often the hardest hit. Of the 262 terrestrial vertebrates experiencing recorded conflict with people, 53 are listed as threatened [4].

Leopards (*Panthera pardus*) are the most widespread large carnivore and can survive across varied land use types outside of protected areas, attributed to their adaptability and opportunistic hunting strategy [5]. However, the leopard has lost 79.4% of its historic home range, resulting in a global population decline and is classified as Vulnerable on the International Union for Conservation of Nature (IUCN) Red List of Threatened Species [2, 6]. Primary threats include habitat loss and fragmentation, prey depletion, and direct persecution [7–11].

Only 20% of South Africa is classified as suitable leopard habitat, and only 25% of that falls within protected areas [12]. Therefore, conserving leopards requires prioritizing and protecting populations outside protected areas [12]. Connectivity between viable habitats is paramount to link subpopulations and ensure gene flow, as well as to enable the species to access mates, food, and habitats [13]. As the habitat available to leopards and other large carnivores continues to decline and becomes increasingly fragmented, human-modified landscapes are becoming more important as both potential habitat and corridors or stepping stones between protected populations [14, 15]. Where leopard movement between core habitats is funnelled through a single or limited pinch points (either created by anthropogenic transformation of land or natural barriers to movement), conservation strategies are required to avoid severance to connectivity [13].

Leopard studies in South Africa have predominantly focused on conservation areas and highly suitable habitats, leaving an exigency for research investigating leopard subpopulations outside of protected areas [16]. In recent years studies have started to address this knowledge gap [e.g. 17–19]. In the Western Cape province of South Africa, research on leopards is steadily emerging from study sites outside of protected areas or crossing the threshold between protected and non-protected land [20–24]. Threats to leopards in a mixed land-use, overlap substantially with those experienced as edge effects in protected areas or more generally within poorly managed protected areas [9, 25]. Understanding how leopards persist in mixed land-uses is important for future leopard conservation and policy outcomes given the threats to leopards in protected areas [26, 27] and the importance of shared landscapes as movement corridors [28].

In the Western Cape province, leopards are the last free-roaming large predator, have comparatively large home ranges and occur at lower densities compared to populations in the Savanna biome of South Africa [20–22, 24, 29]. Leopard density estimates in the Western Cape range from 0.18 per 100 km^2^ to 1.89 per 100 km^2^ [22, 30], with the lowest estimates from small-scale studies in the Overberg (S1 Table in S1 File).

The Western Cape has experienced an increase of approximately 1 million people in the last decade [31] and this trend is predicted to continue, mainly linked to an influx of migrants [32]. Increased human population has been accompanied by an expansion of urban and agricultural land-use in the Western Cape [33], further fragmenting and eroding natural habitats. Agriculture is generally restricted to flatter, lower-lying land, with the Cape Fold Mountains providing a refuge for many plants [34] and animals, including leopard [15]. The mountains also act as natural corridors that allow leopards to move large distances [29, 35, 36]. Due to the ubiquity of human-dominated landscapes within the Western Cape, the majority of predicted ecological corridors for leopards extend along narrow mountainous strips of the Cape Fold Mountains where anthropogenic activity is reduced and factors influencing habitat suitability such as moderate slope and land cover are optimal [37]. Movement through corridors and stepping stones may thus assist gene flow across transformed landscapes, maintaining the genetic viability of isolated populations [38–41].

In addition, human population growth often elicits the unsustainable use of natural resources [42]. For example, hunting bushmeat using wire snares is common in the Western Cape [43, 44]. This illegal hunting method depletes the prey base and leopards are sometimes caught in snares, causing injury or death [18, 45, 46].

The density and occurrence of leopards in mixed-use landscapes depend on the balance between the risks of persecution and the availability of habitat for cover, prey availability, and potential mates [47, 48]. The southernmost population of leopard persists in the Overberg, despite the landscape being heavily modified and transformed by anthropogenic activities in some areas [12, 37, 49]. These characteristics make the Overberg an excellent location to examine how anthropogenic and biological covariates affect leopard density across a fragmented landscape. We use camera trapping in the mixed-use landscape of the Overberg to estimate leopard density and use these data as a first step to explore the potential ecological and anthropogenic factors influencing leopard density. This study provides the first baseline estimate of leopard population density across the Overberg region. Potential corridors and suitable habitat for leopard in the Overberg are severely limited [37]. The factors influencing density can inform management decisions and be used to motivate for improved ecological connectivity strategies for leopards, a keystone species, across the region.

## Materials and methods

### Study area

**Fig 1:**
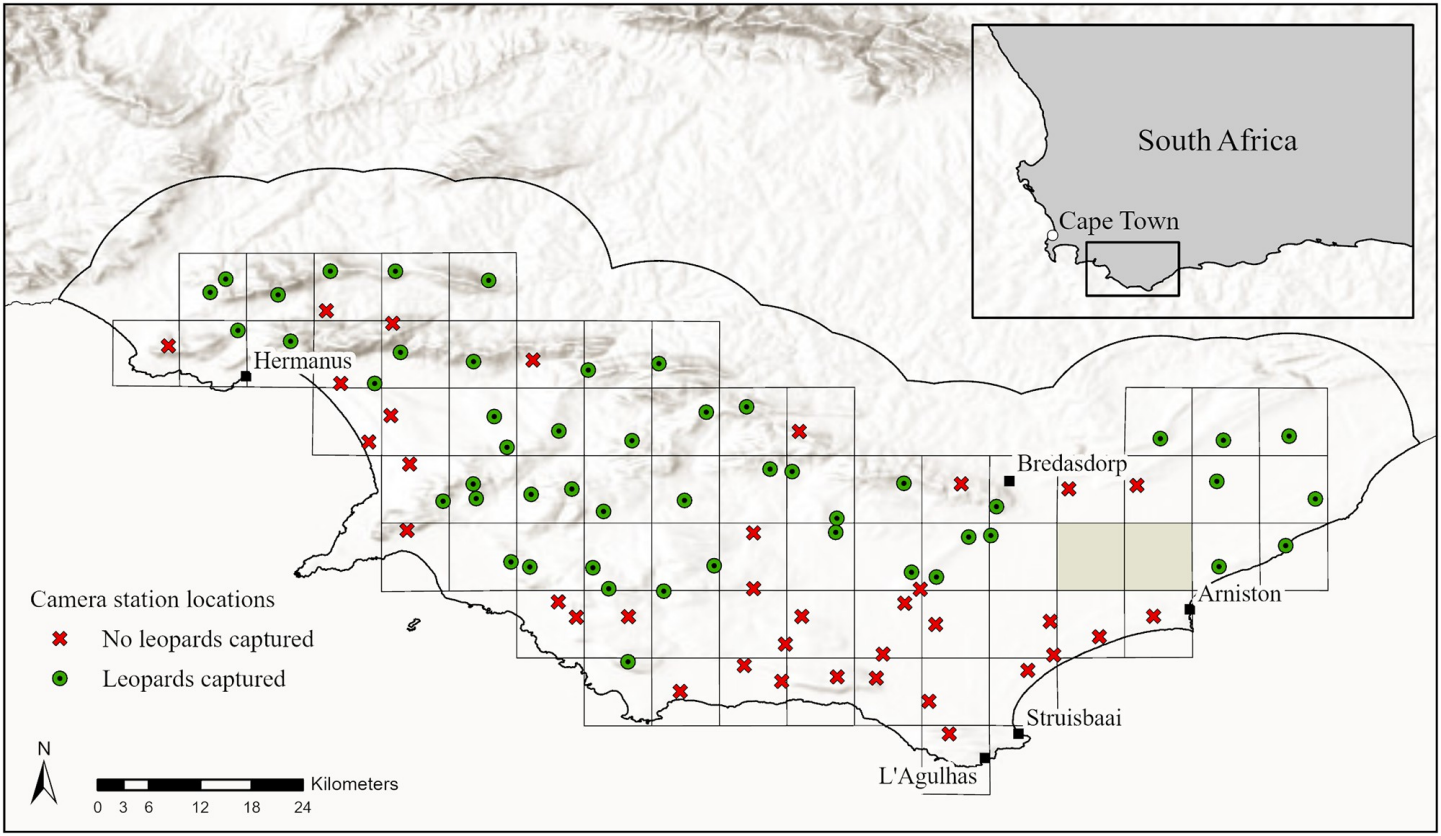
The study area in the Overberg, Western Cape. Each point (green / red) on the map represents the location of a paired camera trap station. Green circles show leopard detections and red crosses show leopard non-detections. The black line north of the camera traps shows the terrestrial limit of the study areas which includes a 12 km buffer zone around the northernmost traps. To the south the coastline of the Indian Ocean serves as a hard boundary. A 50 x 50 km grid was superimposed over the study area with at least one pair of camera traps placed in each grid block (n = 71 blocks; two blocks entirely comprised of tilled crop fields were excluded (shaded in grey) as these were assumed uninhabitable by leopards). The map was created using ArcGIS^®^ software by Esri. ArcGIS^®^ and ArcMap^™^ are the intellectual property of Esri and are used herein under license.

This study was carried out in the southernmost region of the Western Cape and covers an area of approximately 3,500 km2, stretching from the Bot River Estuary in the west to the De Hoop Vlei in the east (Fig 1). The Overberg is considered the breadbasket of the Western Cape with extensive and intensive agricultural activity ranging from cereal crops to vineyards and fruit trees [50]. Land uses and type include agriculture, livestock farming, tourism, and natural fynbos vegetation. The study area encompasses national and provincial protected areas, private protected areas, farmland, and small urban residential settlements.

Annual rainfall in the region averages 460–600 mm and peaks during the austral winter months. Average monthly temperatures range from 8 °C to 27 °C with an annual average of 16 °C (Climate-Data.org). The topography varies from 1,300 m (Cape Fold Mountains) to sea level along the southern coast of South Africa. There are 17 types of vegetation in the study area, of which 10 are threatened (six of which are critically endangered) [51, 52] (S2 Table in S1 File).

### Camera trap survey

A camera trap survey consisting of 86 paired camera stations was active for 160 days (19 August 2021 to 25 January 2022). Sufficient spatial recaptures of individuals are essential for precision and accuracy in density estimates [53], but attaining these within a short time period can be challenging for leopards in the Cape, an elusive species with large home ranges, prompting many Eastern Cape and Western Cape leopard studies to adopt a longer survey duration [e.g. 17, 20, 24]. Due to the leopard’s slow life history, this study’s sampling duration does not jeopardise the validity of results [54; S1 Fig in S1 File]. Camera spacing was based on the minimum home range of a female leopard in the Western Cape [55]. This figure is smaller than recorded female leopard home ranges within or bordering the study area, e.g. De Hoop Nature Reserve = 66.4 km^2^ and Hermanus mixed landscape = 86.7 km^2^ [21].

Camera stations consisted of paired Cuddeback C1 Strobe Flash 1279 trail cameras set at a height of 30–40 cm on opposite sides of a pathway to obtain an image of both the left and right flanks of animals. Camera locations were chosen to maximise leopard detections, and were placed on least resistance pathways (e.g., roads, hiking trails, game trails), ‘natural funnels’ created by the local topography, and where there was signs of leopard presence including scat, spoor, and tree markings. At six-week intervals the camera traps were serviced to maintain functionality, and vegetation was cleared in front of each camera.

### Data analysis

Only adult leopards were included in the analysis. Individual leopards were identified by their unique pelage pattern on the left and right flanks using the program Hotspotter [56, 57]. An independent observer then verified and confirmed the identified individuals. The sex of a leopard was assigned only when images provided clear evidence of either testes or a dewlap [58]. A small number of individuals were recorded only by un-matched left or the right flank images. The total number of individuals represented by only left flanks and only right flanks were counted. The un-matched flank side with least number of individuals recorded was removed to avoid false recaptures. A sampling occasion was defined as a 24 hour period beginning and ending at midday at each camera station [59], and a threshold of 60 minutes was used to temporally distinguish independence of unique leopard photo-capture events.

We estimated leopard density using a Bayesian spatial capture-recapture (SCR) approach. Leopards exhibit sex-specific differences in space-use and behaviour, with the home range of a single adult territorial male overlapping with smaller home ranges of several females [60]. Location of cameras is also a likely source of capture heterogeneity [20]. We first implemented the simpler model p0(sex).σ(sex), where p0 denotes the probability of capture when the distance between the animal’s activity centre and the camera is zero and sigma is the ranging scale parameter, and assessed model fit in the program JAGS (Just Another Gibbs Sampler) accessed through the program R, version 4.0.4 [61] using the package RJAGS (http://mcmc-jags.sourceforge.net). In data augmentation, we set M to 200 –larger than the largest possible population size (i.e., the number of activity centres). Buffer sizes around the trapping grid were plotted to test for density stabilisation [62]. The habitat mask was discretised into 550 m x 550 m (0.3 km^2^) cells, and we included a buffer of 12 km beyond the outermost camera stations and excluded cells with centroids located in pixels classified as water (Fig 1). The 12 km buffer ensured that leopards with home range centres located outside of the study area had a low probability of being detected and results were not biased [63].

We used the centroids of capture locations of individual animals caught as the starting values for activity centres, ensuring these occurred in suitable habitat as defined in the habitat mask. We implemented three Markov Chain Monte Carlo (MCMC) chains with 60,000 iterations, a burn-in of 1,000, and a thinning rate of 10 to ensure an adequate number of iterations to characterise the posterior distributions. We checked chain convergence using the Gelman-Rubin statistic [64], R-hat, which compares between and within chain variation. R-hat values below 1.1 indicate convergence [65]. The approach of Royle et al. [66] was used for the model goodness-of-fit test, calculating three statistics, all using Freeman-Tukey discrepancies: individual animal by camera-station capture frequencies, aggregating the binary daily capture data by animals and camera-stations (FT1); individual animal capture frequencies, aggregating for each animal (FT2); and camera-station animal capture frequencies, aggregating for each camera-station (FT3). We also implemented the model p0(sex+trap).σ(sex) if the simpler model did not fit to the data. We generated posterior distributions of adult male, adult female, overall population density, and male and female ranging parameters from the model.

For leopard density surface modelling, we used maximum-likelihood SCR density models with covariates in the package ‘secr’ [63] in R, version 4.1.3. [67]. We examined bottom-up environmental factors: Normalised Difference Vegetation Index (NDVI) [68]; Elevation based on 30 m resolution Digital Elevation Model [69]; Terrain Ruggedness Index (calculated from elevation in QGIS); and Vegetation Bioregion [70]. Top-down anthropogenic factors included: protected land in the study site [71]; human population density [72]; and major land-use [73]. The 63-classified land-use types in the study area were divided into one of three categories: urban (e.g. artificial waterbodies, residential, commercial), agricultural, and natural (S3 Table in S1 File). The covariates were incorporated into each cell of the habitat mask. Spatially continuous variables were averaged using inverse distance weighted means across cells surrounding the habitat cell [27]. The weight assigned to each pixel surrounding the habitat cell within an animal’s home range was relative to the probability that a leopard will be detected within that cell [27]. We included squared predicators for elevation, terrain ruggedness, and NDVI to account for situations where leopards may prefer either very low or very high extremes.

Leopard density was also estimated using a maximum likelihood approach in the package ‘secr’ [63] in R, version 4.1.3. [67] for comparison with previous estimates from the region. After considering previous leopard studies [60, 74] and testing the fit of various detection functions, a hazard half-normal detection function was used. Sex was used as a covariate on λ_0_ (expected number of detections at distance zero between the camera and the animal’s activity centre) and σ (spatial rate of decay). To avoid models converging on local maxima, models were rerun with the starting values as the output of previous runs. Relative statistical support was selected using Akaike’s Information Criterion corrected for small sample sizes (AICc). Models that had AICc scores below seven relative to the best performing model were considered to show adequate support [75]

## Results

The 86 camera stations were active for 13,500 camera trap nights. Leopards were detected at 50 of the 86 camera stations (58%). Independent captures (a sampling occasion is 24 hours) of leopards totalled 221 occasions and included 25 individual adult leopards (14 females, 9 males, and 2 unsexed individuals). Seven juveniles and three unidentified individuals were captured on cameras but excluded from further analysis. In the first 100 days of the survey, 92% of individual adult leopards were detected (S2 Fig in S1 File).

The buffered study area encompassed roughly 4,650 km2, of which 862 km2 (19%) was comprised of patches of protected land. Natural land made up most of the study area (63%) followed by agricultural (35%) and urban (2%). Camera traps were placed on eight different vegetation types (S2 Table in S1 File). Overberg Sandstone Fynbos had the most camera trap sites (n = 38) followed by Agulhas Limestone Fynbos (n = 15). Elevations ranged from a maximum of 1,150 m to sea level at the coast. Terrain ruggedness index varied from level areas (TRI = 0 m) to moderately rugged areas (TRI = 485 m). The human population density ranged from 0 to 17,500 people per km2 in the coastal town of Hermanus. Terrain ruggedness index is derived from Elevation and as such were found to be highly correlated (R = 0.93) but both variables were retained because they do not occur in the same model. No other continuous variables were correlated more than R >0.4.

**Fig 2:**
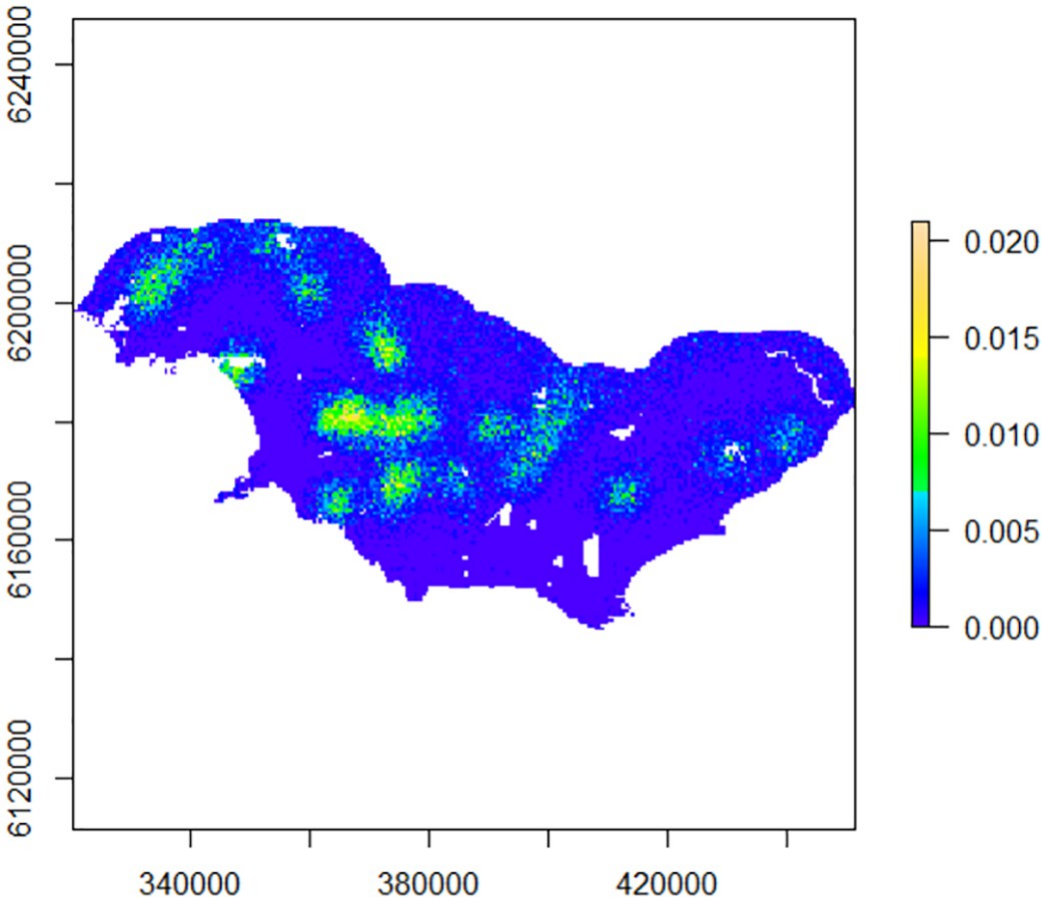
Predicted density of individual leopards (individuals per km^2^) ascertained from the Bayesian SCR model. The southern boundary is the coastline of the Indian Ocean.

**Fig 3:**
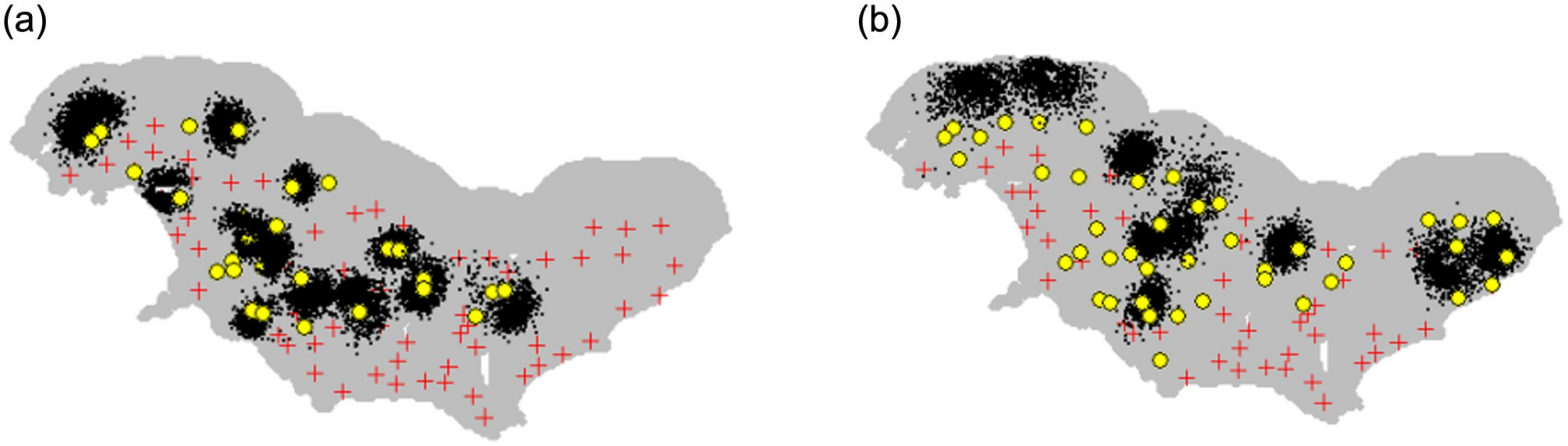
Activity centre posterior distributions for adult female (left) and adult male (right) leopards in the Overberg, Western Cape. Black dots represent uncertainty in animal activity centres. Yellow circles represent capture locations and red crosses indicate camera trap locations.

The Bayesian p0(sex+trap)sigma(sex) model fitted well to the data (FT1, FT2, FT3 P = 0.3–0.5), and R-hat values for all model parameters were below 1.1. Leopard population density was estimated as 0.64 leopards per 100 km^2^ (CV: 7%, 95% CI: 0.55–0.73) and ranged across the study area with the lowest densities near the coast (Fig 2). Female sigma was estimated at 3.42 km (95% CI: 2.99–3.87 km) and male sigma was 6.92 km (95% CI: 5.85–8 km). Females activity centres were more centralised while male activity centres were mostly situated on the edges of the study area (Fig 3).

Using a maximum likelihood approach, the best performing model, MD6, includes the covariate sex on the detection parameter σ, and a combination of sex, vegetation type and the interaction on λ_0_. AICc scores revealed that MD7, MD5 and MD1 all have support (dAICc < 7; Table 1). MD6, which accounted for more than 80% of the weighting, predicted a density of 0.64 leopards per 100 km2 (95% CI: 0.43–0.94) across the buffered study area (4,575 km2). Female σ was estimated at 3.18 km (95% CI: 2.85–3.56 km) with male σ substantially greater, 7.14 km (95% CI: 6.31–8.09 km). The encounter rate of males and females (λ_0_) was 0.0140 (95% CI: 0.0088–0.0222) and 0.0245 (95% CI: 0.0148–0.0404), respectively. MD7 showed adequate support and therefore, sex was used as a covariate on σ and λ_0_ for inhomogeneous models. MD7 was chosen over MD6, as the model contains fewer parameters, and therefore the inhomogeneous density models performed better with only sex as a covariate on σ and λ_0_.

**Table 1:** Homogenous density models with different detection parameters on *σ* and *λ*_0_. Vegetation type represents the vegetation the camera station was placed in. Sex is individual leopards captured (male, female, or unsexed). Asterix indicates a third term, which is the interaction term in addition to the main effects of the variable, a colon is simply the interaction term. The best-performing model parameters were used for inhomogeneous density models.

Model	Predictor variables on *λ*_0_	Predictor variables on *σ*	N parameters	AICc	dAICc	AICc weight
MD6	Vegetation Type * Sex	Sex	10	738.446	0	0.8004
MD7	Sex	Sex	6	753.725	4.23	0.0965
MD5	Vegetation Type: Sex	Sex	11	739.301	5.45	0.525
MD1	Vegetation Type + Sex	Vegetation Type + Sex	10	743.972	5.53	0
MD4	Vegetation Type + Sex	Sex	8	753.29	8.13	0
MD3	Vegetation Type * Sex	Vegetation Type * Sex	14	731.67	19.51	0
MD2	Vegetation Type: Sex	Vegetation Type: Sex	16	733.166	47.01	0
MD0	1	1	4	864.683	112.52	0

All individual covariate density models showed some support. Elevation, terrain ruggedness, and NDVI showed high support (dAICc < 2); with human density and protected status showing intermediate support (dAICc < 7) and land-use category and vegetation bioregion the least support (Table 2). The best performing model, which accounted for almost 40% of the weight, included elevation.

**Table 2:** Performance of predictors for drivers of leopard density in the Overberg expressed as weighed support. Elevation represents height above sea level in meters at a resolution of 30 meters; Terrain ruggedness is a measure of the difference in elevation of a cell and the eight surrounding cells; Protected status is a binary variable of whether an area is protected area or not; NDVI is a proxy for vegetation productivity; Human density represents the human population density in each cell of the habitat mask; Land-use is a categorical variable of land as either natural, agricultural, or urban. Vegetation bioregion is a categorial variable indicating the six bioregions represented in the survey area.

Covariate	Model	N parameters	AICc	dAICc	AICc weight
Elevation	D ~ Elevation + I(Elevation^2)	7	759.214	0	0.3945
Terrain ruggedness	D~TRI + I(TRI^2)	8	760.847	1.63	0.1744
NDVI	D~NDVI + I(NDVI^2)	8	761.117	1.9	0.1523
Human density	D ~ Pop.Density	7	761.578	2.36	0.121
Protected status	D~ Status	7	761.843	2.629	0.1099
Land-use	D ~ land use	8	766.396	7.18	0
Vegetation bioregion	D ~ bioregion	12	787.289	28.075	0

**Fig 4:**
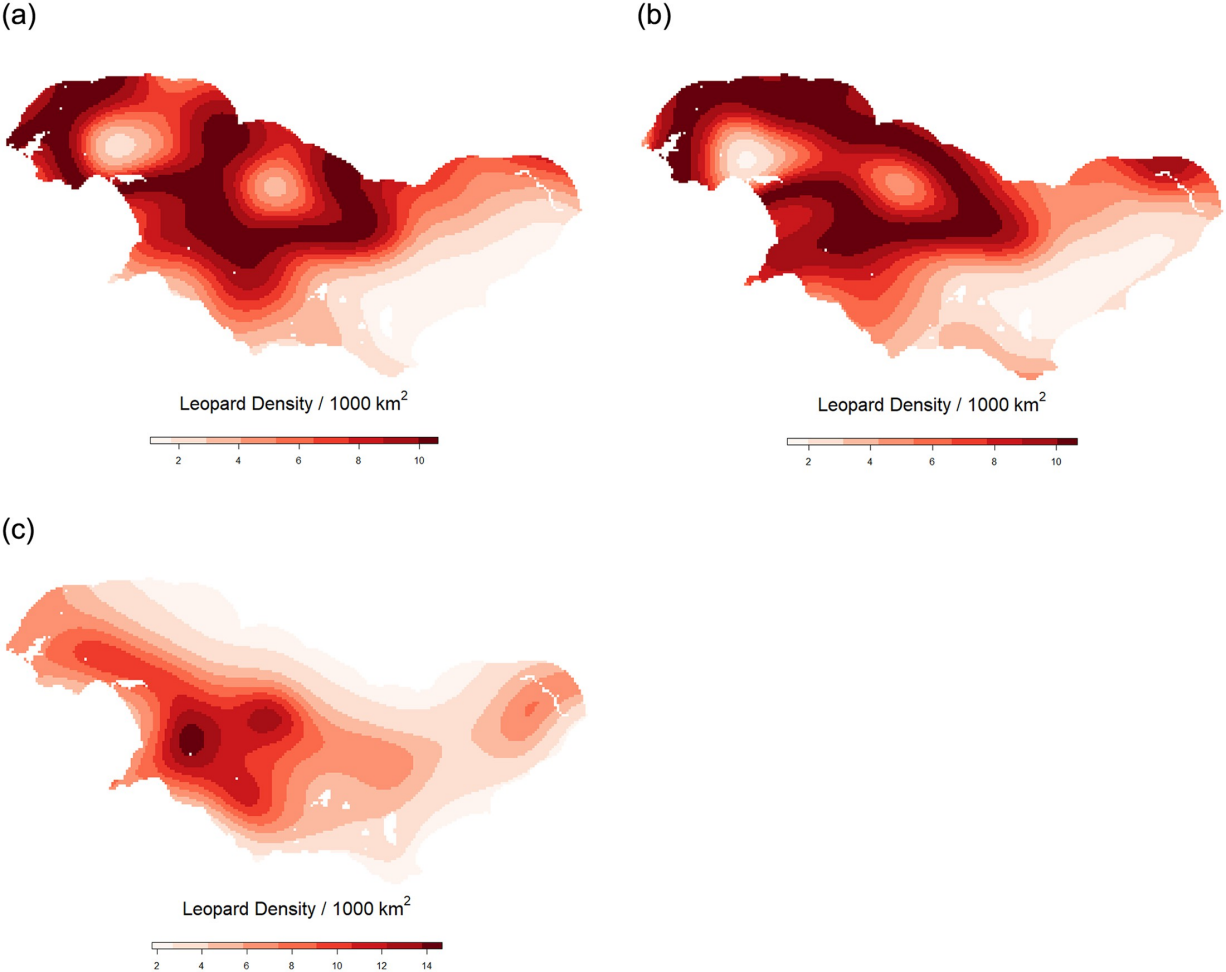
Heat maps showing how leopard density responds to the best performing predictors (dAICc < 2; Table 2) within the surveyed area. A: Elevation model; B: Terrain ruggedness model; C: NDVI model.

The coefficient of elevation (β = 0.58) indicates a positive relationship between elevation and leopard density (Table 3 and Fig 4). Terrain ruggedness was also positively correlated with density, but protected areas and NDVI had a negative relationship with density (β = -0.53, -0.56). Leopard density also showed a negative relationship with levels of human population density. Density showed a positive effect for natural land-use and a slightly positive effect for agricultural land. Squared predictors indicate the effect on the extremes of predictors, therefore extreme elevations and ruggedness, are associated with lower leopard densities than intermediate values while extremes of vegetative productivity had a positive influence (Table 3).

**Table 3:** Performance of predictors for drivers of leopard density in the Overberg expressed as a positive or negative influence. Elevation represents height above sea level in meters at a resolution of 30 meters; Terrain ruggedness is a measure of the difference in elevation of a cell and the eight surrounding cells; Protected status is a binary variable of whether an area is a protected area or not; NDVI is a proxy for vegetation productivity; Human density represents the human population density in each cell of the habitat mask; Land-use is a categorical variable of land as either natural, agricultural, or urban; Vegetation bioregion is a categorial variable indicating the six bioregions represented in the survey area. Elevation, Terrain ruggedness, and NDVI models all include *x*2 quadratic term to represent the effect of extreme value.

Predictor	Beta estimate	Standard error	P value
Elevation	0.58	0.28	X>0; P = 0.98
Elevation^2^	-0.56	0.29	X<0; P = 0.97
Terrain ruggedness	0.58	0.28	X>0; P = 0.98
Terrain ruggedness^2^	-0.47	0.28	X<0; P = 0.95
Protection status	Protected: -0.53	Protected: 0.84	X<0; P = 0.74
NDVI	-0.56	0.29	X<0; P = 0.97
NDVI^2^	0.08	0.29	X>0; P = 0.61
Human density	-0.25	0.36	X<0: P = 0.76
Land-use	Natural: 0.22	Natural: 8.47	N/A
	Agricultural: 0.02	Agricultural: 8.55	
Vegetation bioregion	East Coast Renosterveld: 10.08	East Coast Renosterveld: 51.11	N/A
	South Strandveld: -9.67	South Strandveld: 0.06	
	South Coast Fynbos: 10.39	South Coast Fynbos: 51.11	
	Seashore Vegetation: -8.90	Seashore Vegetation: 0.21	
	South West Fynbos: 10.92	South West Fynbos: 51.11	
	Zonal & Intrazonal Forests: 3.34	Zonal & Intrazonal Forests: 204.2	

## Discussion

### Density

This study’s estimate of 0.64 leopards per 100 km2 falls in the middle of density estimates for the Western Cape region (S1 Table in S1 File). A 2011 survey which covered a smaller section of the Overberg and identified eight leopards at only 28% of trap sites, estimated 0.17 leopards per 100 km2 using a maximum likelihood approach using secr and 0.69 leopards per 100 km2 using a Bayesian approach employing SPACECAP, a commensurate estimate to this survey [22]. Variation between this study’s density estimate and the previous estimate in the Overberg [22] may be the result of differing survey area, recapture rates, survey length, and fluctuations in the leopard population over time amongst other factors. Leopard density estimates in other areas of the Western Cape range from 0.18 leopards per 100 km2 in De Hoop Nature Reserve (which borders this study on the eastern boundary but covered a much smaller area using a more densely and closely configured camera trapping array) [30] to 1.89 leopards per100 km2 in Langeberg [22] (S1 Table in S1 File). Leopard densities in northern and eastern South Africa [76–78] are generally higher than density estimates for leopards in the Western Cape [15, 20, 22, 24]. These areas with higher leopard densities often fall within more suitable leopard habitat and within protected areas [12, 77].

Density estimations are dependent on what the authors classify as available leopard habitat and this should be considered when densities are compared in a region. For example, this study included natural, agricultural, and urban areas while Amin et al. [20] considered leopard density in natural vegetation only and excluded transformed agricultural areas and urban areas from the model space. As leopards in the Western Cape have been found to occupy natural habitats at higher density than transformed land [12, 22, 37], densities considering only natural land will in most instances lead to higher estimates.

### Drivers of leopard density in a mixed-use landscape

Elevation, terrain ruggedness, and vegetation productivity most strongly influenced leopard density in the Overberg. Elevation, which was correlated to the terrain ruggedness index, may serve as a proxy for leopard refugia with high relative elevation invariably precluding urban and agricultural land-use [12, 15]. Leopards are highly mobile and agile climbers and thus terrain ruggedness does not impede their movement but rather provides them with cover to better ambush their prey [47]. Rugged areas are also not suitable for agricultural or urban land-uses and are therefore invariably less transformed and more suitable for wildlife [79, 80]. Elevation was also found to be an important driving factor for density in the semi-arid Little Karoo [15], with low elevations used for intensive agricultural and urban development, which effectively excluded leopards. Research on both baboons (*Papio ursinus*) and caracal (*Caracal caracal*) in a mixed land-use region of the Western Cape revealed that both species prefer low lying land but are displaced to higher elevations by anthropogenic land-uses [81, 82]. In the Overberg, most low elevation land is flatter and more suitable for development, which may explain why the effects of elevation were more marked in this region than in the Little Karoo [15].

Both NDVI [12] and terrain ruggedness [83, 84] have been used as proxies for prey catchability / vulnerability. NDVI and terrain ruggedness influence the visibility of both prey and predators; the probability that individuals will move along paths/roads, i.e. when surrounding vegetation is dense, they are more likely to use these features; the amount of prey a vegetation type supports; and the cover, by both the terrain and/or the vegetation, afforded to leopards when stalking prey [47]. In a study examining 27 protected areas of South Africa, NDVI was positively correlated with leopard density [74]. Yet in the Overberg leopard density was negatively influenced by higher NDVI. However very high NDVI did have a slightly positive effect on density possibly serving as a refuge for leopards in this mixed-use landscape and thus suggesting a non-linear relationship between density and NDVI. A similar negative correlation between leopard density and NDVI was found by Smyth [85] in an area that had historically high levels of anthropogenic impact. Here it was argued that positive bottom-up effects associated with higher NDVI levels were swamped by chronic levels of top-down anthropogenic impacts.

Bottom-up factors (habitat suitability and catchable prey) are the principal drivers of apex predators’ movements in natural systems [86]. However, in anthropogenically dominated systems humans have a disproportionate influence on ecosystems, from top-down regulation (direct persecution) to bottom-up regulation (depletion of prey bases) [87–89]. Human population density is thus an important variable influencing the density of leopards [60, 90]. Land-use is also important, with protected areas providing a refuge for both prey and predators relative to agricultural and urban land-uses [9, 91]. Contrary to expectation, human density, protected status, and land use did not strongly influence leopard density in the Overberg.

The importance of protected areas for leopards is well established [12, 27, 48], however, large felid density can be similar or even higher on agricultural land compared to nearby protected areas [e.g. 92, 93]. In this study leopard density was higher outside of protected land (Fig 2). Leopards were only captured at two of the eleven camera sites in Agulhas National Park, the largest formally protected area in the survey area, while a recent leopard survey in De Hoop Nature Reserve, a 340 km2 protected area on the boundary of the study area, revealed a very low density of leopards (0.18 per 100 km2) [30]. In the absence of any clear evidence of direct or indirect negative human impacts in either protected area, the most parsimonious explanation for low leopard numbers within these protected areas is that the environmental variables therein are largely unsuitable for leopards. Only 30% of protected areas in the Western Cape are comprised of suitable leopard habitats [12], with Maxent modelling revealing that the Agulhas National Park, a low lying area, is not considered suitable habitat for leopards [22, 37].

However, this study did indicate that leopard density was higher in low intensity use agricultural land than urban land—showing a clear negative cascade from natural to urban land-use. Although leopards can be attracted to agricultural areas due to the potential availability of both domestic and synanthropic food sources [94–96], results reveal higher densities in natural vegetation where natural prey are both more abundant and comprise a higher proportion of leopard diet [79, 97]. Unfortunately, it was not possible to incorporate covariates for prey abundance / density and domestic animal / human presence as they are session-level covariates and hence only once this study is repeated can they be included. Both variables have been shown to be important drivers of density within protected areas [62, 74, 98] and thus their omission is a weakness of the current study.

### Conservation recommendations

With large home ranges which span up to 910 km2 in the Western Cape [29] and the ability to generate public interest to a greater extent than many other species [99], leopards can act as umbrella and flagship species for ecosystem conservation. The opportunity to use the leopard as a flagship species to open conservation discussions and instil positive change was incorporated into the study design. Running a camera trap survey in a mixed-use landscape required considerable interactions with private landowners. By explaining the research objectives and sharing wildlife images from this survey with individual landowners, the people living in this shared landscape were educated on the threats to, and benefits associated with retaining large predators and other wildlife in the landscape. Alongside these direct interactions with landowners, ecology and conservation lessons were shared with learners at local schools, and messages were fortified and given local relevance by incorporating images and research results from this study. Environmental education is critical to improving public support and tolerance for wildlife outside of protected areas [100, 101] and is an important, yet sometimes overlooked, tool in effective species conservation [102–104]. While the impact of outreach was unquantified in this study, it is suggested that repeated camera trap surveys aim to do more than merely monitor changes in the population over time but offer the opportunity for regular educational drives and partnerships with landowners that may be of significant benefit to biodiversity conservation in the Overberg. It is here that NGOs provide a critical service to conservation, sourcing the funding required for long term effort outside of protected areas where resources are limited and not under the governance of protected area managers. It is hoped that a better understanding of the biodiversity living on private land will promote land-use practices that are less detrimental to both the environment and the biota that depend on it.

The large spatial requirements of leopard in the Cape emphasise the role leopard can play in conservation as an umbrella species but also places extra pressure on protecting and expanding connectivity for the species, especially across tracts of modified land such as in the Overberg. Connectivity, particularly in small populations, is important for finding mates, allowing dispersal, and ensuring genetic diversity in the population [38–40]. The variation in density of leopards throughout the Western Cape reveals potential source and sink populations that can ensure stable regional populations over time. However, this will require persistent movement corridors to be retained and possibly created where movement is impeded [37]. As natural habitats and rugged higher elevations harbour the highest leopard densities, the Cape Fold Mountains offer a significant natural corridor for the region. Areas displaying favourable characteristics for leopard density (in terms of elevation, terrain ruggedness and vegetation productivity) are found in pockets across predominantly western sections of the Overberg region. A conservation priority for leopard survival in the region should be the creation and safeguarding of corridors to enable movement between these areas and the Cape Fold Mountains in the west and other core habitats such as De Hoop Nature Reserve in the east.

Improved connectivity and the persistence of leopards outside of protected areas provide a critical buffer for stochastic events such as extensive fires and droughts that may both be exacerbated by climate change [105–108]. The Western Cape is predicted to become hotter and drier, and the Cape Fold Mountains, which run in an East-West direction across most of the southern Western Cape provide access to cooler south-facing slopes that are predicted to be critical refugia for a range of wildlife including leopards and species they prey upon [109, 110].

## Conclusions

Protected areas are becoming increasingly fragmented from each other with a hardening of their edges and do not fully shield species from anthropogenic risks [74, 111, 112]; this limits their ability to maintain viable populations of large carnivores without adopting an intensive metapopulation approach with assisted dispersals through translocations. Unlike large carnivores with greater rigidity in their ecological requirements, leopard can persist and even thrive on land outside of protected areas, yet little is known about leopard density and factors impacting density in heavily modified and fragmented landscapes in South Africa [12]. In this study leopards were detected throughout a mosaic of different land-uses, with higher densities outside of protected land.

High leopard densities outside of protected areas is a phenomenon more commonly associated with countries like India (~10 leopards per 100 km^2^; [95]) where tolerance for large carnivores is high (and enforced by law)–despite their diets consisting mainly of livestock and pets [113]. There are few reports of negative leopard impacts in this region of the Overberg, although livestock depredation is occasionally reported to local NGOs and CapeNature, the provincial conservation body (unpublished records). While tolerance of predators by farmers in South Africa is generally low [114], it is unlikely that leopards are being actively persecuted in this region. Due to the Overberg’s high fragmentation and limited connectivity for leopard movement, coupled with a low leopard density, the leopard population is highly vulnerable to increases in anthropogenic threats. Conflict incidents between people and predators must be continually monitored and if an increase in incidents is detected, interventions to promote coexistence are required without delay. The Overberg is an excellent region in which to promote the tolerance of leopards and considering the importance of elevation and terrain ruggedness for leopard density, to highlight the importance of the Cape Fold Mountains as corridors connecting areas of higher and lower leopard density. These refugia, in conjunction with the persistence of leopards in mixed land-use areas, suggest that the Western Cape may well be a stronghold for leopards moving forward, even if they naturally occur at low densities in this region. Genetic variability will need to be maintained for the population to persist by ensuring the maintenance of natural areas and creation of corridors. It is thus important to engage in long-term monitoring of this and other leopard populations in the Western Cape as a lens on how climate, human population growth, and land-use changes may impact apex predators and their prey outside of protected areas across a range of anthropogenic and natural land uses.

## Supporting information

S1 File (DOCX)
