# Supplementary material for: Leopard density and the ecological and anthropogenic factors influencing density in a mixed-use landscape in the Western Cape, South Africa
Source: PLoS One. 2023 Oct 27;18(10):e0293445. doi: 10.1371/journal.pone.0293445 (PMC10610481; doi:10.1371/journal.pone.0293445)
Supplement: S1 File — (DOCX) [file pone.0293445.s001.docx]

# **Supporting information**

**Leopard density and the ecological and anthropogenic factors influencing density in a mixed-use landscape in the Western Cape, South Africa**

**S1 Table**. **A summary of studies that have estimated leopard density in the Western Cape of South Africa**. Includes the statistical method used (Bayesian models and density estimates are shaded in grey), the region where the study was carried out and the year in which the camera trap survey was run.

| Source | Density  leopards/100 km^2^ | Statistical package | Region in the Western Cape of South Africa | Year of Camera Trap Survey |
| --- | --- | --- | --- | --- |
| This study | 0.64 (95% CI: 0.56 - 0.73) | SCR- Bayesian (JAGS) | Overberg | 2021-2022 |
| This study | 0.64 (95% CI: 0.43 - 0.94) | SCR- Maximum Likelihood (secr) | Overberg | 2021-2022 |
| Hargey 2022 [1] | 0.18 (SE + 0.07) | SCR- Maximum Likelihood (secr) | De Hoop Nature Reserve | 2020 |
| Müller et al. 2022 [2] | 1.53 (95% CI: 1.18–1.89) | SCR- Bayesian (JAGS) | Cederberg Mountains | 2017-2018 |
| Mann et al. (unpublished) | 1.10 (SE + 0.2) | SCR-Bayesian (Bayes) | Little Karoo | 2017 |
| Amin et al. 2022 [3] | 1.69 (95% CI = 1.4–1.99) | SCR- Bayesian (JAGS) | Boland Mountain Complex | 2010-2011 |
| Mann et al. 2020 [4] | 1.26 (SE + 0.25) | SCR- Maximum Likelihood (secr) | Little Karoo | 2011-2012 |
| Devens et al. 2021 [5] | 0.50 (95% CI = 0.39–1.09) | SCR- Maximum Likelihood (secr) | Langeberg | 2012-2013 |
|  | 1.89 (95% CI = 0.89–2.50) | SCR- Bayesian (SPACECAP) |  |  |
|  | 0.38 (95% CI = 0.17–0.87) | SCR- Maximum Likelihood (secr) | Garden Route | 2012-2015 |
|  | 0.96 (95% CI = 0.52–1.49) | SCR- Bayesian (SPACECAP) |  |  |
|  | 0.17 (95% CI = 0.06–0.48) | SCR- Maximum Likelihood (secr) | Overberg/  Agulhas | 2011-2012 |
|  | 0.69 (95% CI = 0.39–1.28 | SCR- Bayesian (SPACECAP) |  |  |

**S2 Table.** **Vegetation types present within the study area grouped by bioregions and the number of camera trap stations present on each vegetation type.** The percentage of sites with leopards captured is the total number of camera stations within a vegetation type and how many of them captured leopards [6].

| Bioregion | Vegetation Type | Threat Status | Number of Camera traps on vegetation type | Percentage of sites with leopards captured(%) |
| --- | --- | --- | --- | --- |
| East Coast Renosterveld | Central Ruens Shale Renosterveld | Critically endangered | 1 | 0 |
|  | Eastern Ruens Shale Renosterveld | Endangered | 0 |  |
|  | Ruens Silcrete Renosterveld | Endangered | 0 |  |
|  | Western Ruens Shale Renosterveld | Critically endangered | 0 |  |
| South Coast Fynbos | Agulhas Limestone Fynbos | Critically endangered | 15 | 60 |
|  | Agulhas Sand Fynbos | Critically endangered | 3 | 0 |
|  | De Hoop Limestone Fynbos | Least concern | 10 | 50 |
| Southwest Fynbos | Elim Ferricrete Fynbos | Endangered | 9 | 67 |
|  | Greyton Shale Fynbos | Near threatened | 0 |  |
|  | Hangklip Sand Fynbos | Critically endangered | 0 |  |
|  | Kogelberg Sandstone Fynbos | Critically endangered | 0 |  |
|  | Overberg Sandstone Fynbos | Least concern | 38 | 68 |
|  | Western Coastal Shale Band Vegetation | Least concern | 2 | 100 |
| Seashore Vegetation | Cape Seashore Vegetation | Least concern | 0 |  |
| South Strandveld | Overberg Dune Strandveld | Endangered | 8 | 25 |
| Zonal & Intrazonal Forests | Southern Afrotemperate Forest | Least concern | 0 |  |
|  | Southern Coastal Forest | Least concern | 0 |  |

**S1 Fig. Individual leopard camera trap detections in the Overberg, South Africa, between 19 August 2021 and 25 January 2022.**

**
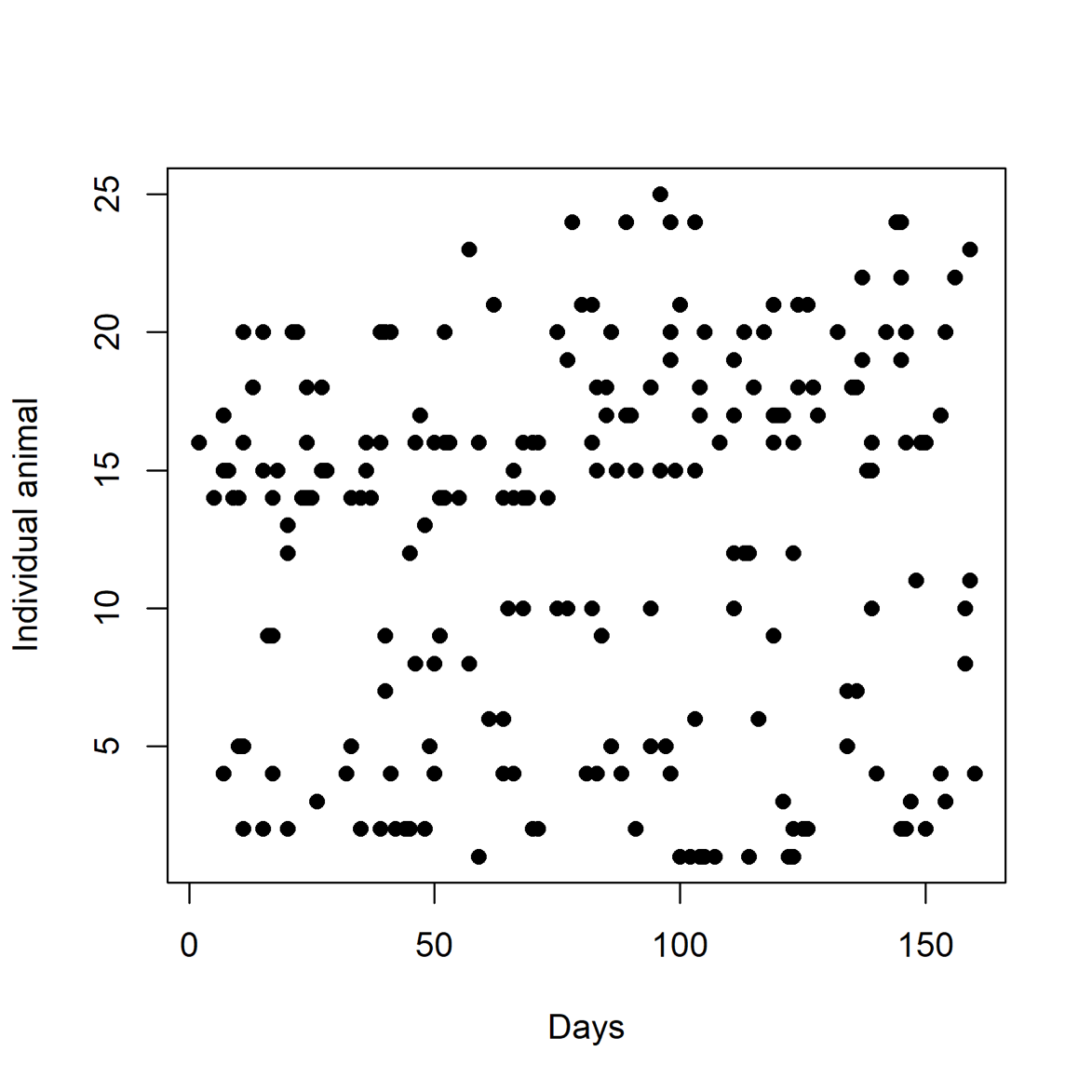
**

**S3 Table.** **Reclassified land-use classes across study area from the South African National Land Cover 2020 Dataset [7].** Columns 1-3 represent original columns from the National dataset, Column 4 is the reclassified land uses used in this study.

| Class_Name | SALCC_1 | SALCC_2 | Land-use class |
| --- | --- | --- | --- |
| contiguous (indigenous) forest | Forested Land | Natural Wooded Land | **Natural** |
| contiguous low forest & thicket | Forested land | Natural Wooded Land |  |
| dense forest & woodland | Forested land | Natural Wooded Land |  |
| open woodland | Forested land | Natural Wooded Land |  |
| contiguous & dense plantation forest | Forested land | Planted Forest | **Agricultural** |
| open & sparse plantation forest | Forested land | Planted Forest |  |
| temporary unplanted (clear-felled) plantation forest | Forested land | Planted Forest |  |
| low shrubland (other) | Shrubland | Shrubs | **Natural** |
| low shrubland (fynbos) | Shrubland | Karoo & Fynbos Shrubland |  |
| sparsely wooded grassland | Grassland | Natural Grassland |  |
| natural grassland | Grassland | Natural Grassland |  |
| natural rivers | Waterbodies | Natural Waterbodies |  |
| natural estuaries & lagoons | Waterbodies | Natural Waterbodies |  |
| natural ocean & coastal | Waterbodies | Natural Waterbodies |  |
| natural pans (flooded @ observation times) | Waterbodies | Natural Waterbodies |  |
| artificial dams (including canals) | Waterbodies | Artificial Waterbodies | **Agricultural** |
| artificial sewage ponds | Waterbodies | Artificial Waterbodies | **Urban** |
| artificial flooded mine pits | Waterbodies | Artificial Waterbodies |  |
| herbaceous wetlands (currently mapped) | Wetlands | Herbaceous Wetlands | **Natural** |
| herbaceous wetlands (previously mapped) | Wetlands | Herbaceous Wetlands |  |
| natural rock surfaces | Barren Land | Consolidated |  |
| dry pans | Barren Land | Consolidated |  |
| eroded lands | Barren Land | Unconsolidated | **Agricultural** |
| coastal sand & dunes | Barren Land | Unconsolidated | **Natural** |
| bare riverbed material | Barren Land | Unconsolidated |  |
| other bare | Barren Land | Unconsolidated |  |
| cultivated commercial permanent orchards | Cultivated | Permanent Crops | **Agricultural** |
| cultivated commercial permanent vines | Cultivated | Permanent Crops |  |
| commercial annual crops pivot irrigated | Cultivated | Temporary Crops |  |
| commercial annual crops non-pivot irrigated | Cultivated | Temporary Crops |  |
| commercial annual crops rain-fed / dryland | Cultivated | Temporary Crops |  |
| subsistence / small-scale annual crops | Cultivated | Temporary Crops |  |
| fallow land & old fields (trees) | Cultivated | Fallow Lands & Old Fields |  |
| fallow land & old fields (bush) | Cultivated | Fallow Lands & Old Fields |  |
| fallow land & old fields (grass) | Cultivated | Fallow Lands & Old Fields |  |
| fallow land & old fields (bare) | Cultivated | Fallow Lands & Old Fields |  |
| fallow land & old fields (low shrub) | Cultivated | Fallow Lands & Old Fields |  |
| residential formal (tree) | Built-up | Residential | **Urban** |
| residential formal (bush) | Built-up | Residential |  |
| residential formal (low veg / grass) | Built-up | Residential |  |
| residential formal (bare) | Built-up | Residential |  |
| residential informal (tree) | Built-up | Residential |  |
| residential informal (bush) | Built-up | Residential |  |
| residential informal (low veg / grass) | Built-up | Residential |  |
| residential informal (bare) | Built-up | Residential |  |
| village scattered (bare & low veg/ grass combo) | Built-up | Village |  |
| village dense (bare & low veg / grass combo) | Built-up | Village |  |
| smallholdings (tree) | Built-up | Smallholdings |  |
| smallholdings (bush) | Built-up | Smallholdings |  |
| smallholdings (low veg / grass) | Built-up | Smallholdings |  |
| smallholdings (bare) | Built-up | Smallholdings |  |
| urban recreational fields (tree) | Built-up | Urban Vegetation |  |
| urban recreational fields (bush) | Built-up | Urban Vegetation |  |
| urban recreational fields (grass) | Built-up | Urban Vegetation |  |
| urban recreational fields (bare) | Built-up | Urban Vegetation |  |
| commercial | Built-up | Commercial |  |
| industrial | Built-up | Industrial |  |
| roads & rails (major linear) | Built-up | Transport |  |
| mines: surface infrastructure | Mines & Quarries | Surface Infrastructure |  |
| mines: extraction pits, quarries | Mines & Quarries | Extraction Sites |  |
| mine: tailings and resource dumps | Mines & Quarries | Waste & Resource Dumps |  |
| land-fills | Mines & Quarries | Waste & Resource Dumps |  |
| fallow land & old fields (wetlands) | Cultivated | Fallow Lands & Old Fields | **Agricultural** |

**S2 Fig. Accumulation curve for leopard detection on camera traps in the Overberg, South Africa, between 19 August 2021 and 25 January 2022.**


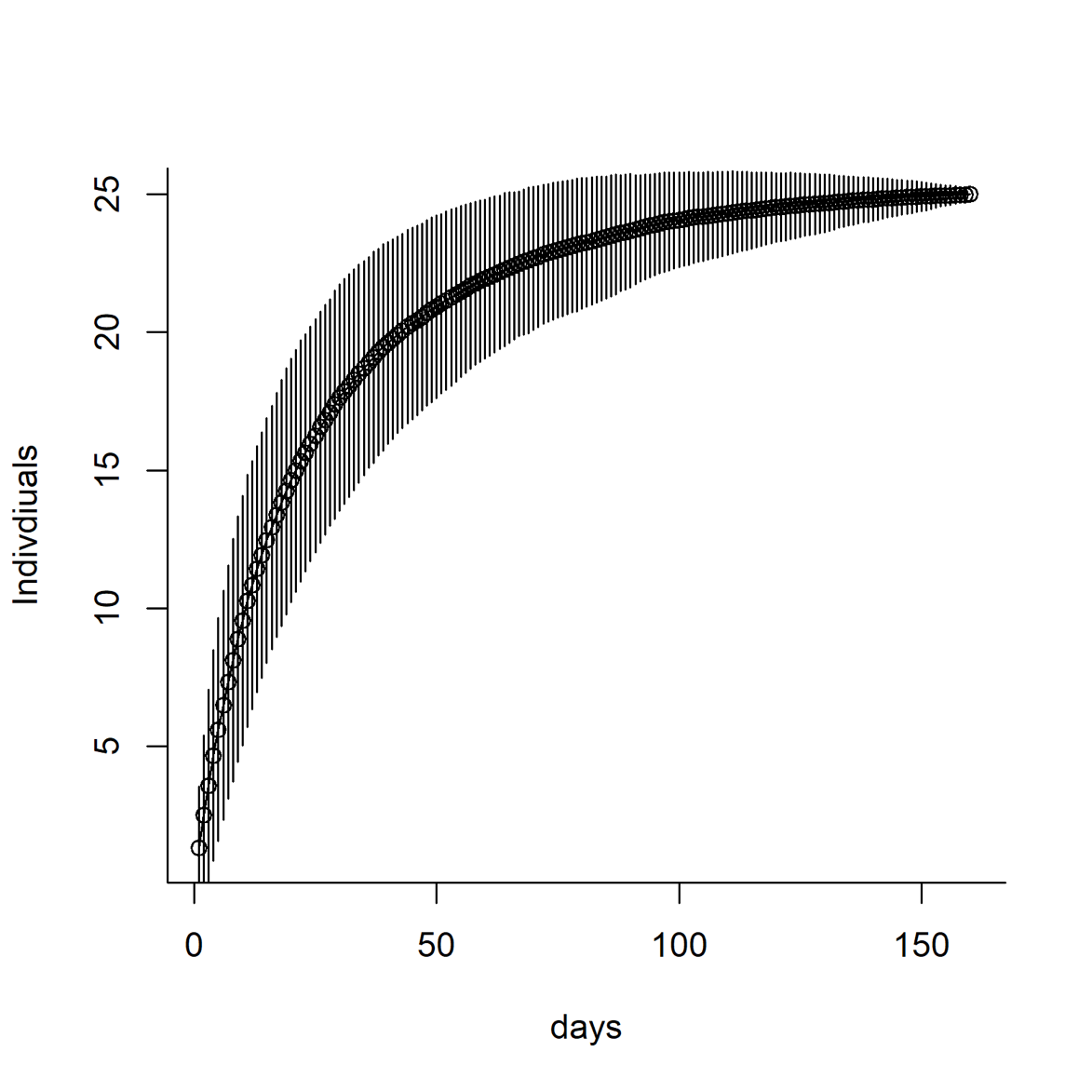


# **References**

1. Hargey A. Estimating leopard density in a coastal protected area of the Western Cape, South Africa [MSc]. Cape Town: University of Cape Town; 2022.

2. Müller L, Briers-Louw WD, Seele BC, Stefanus Lochner C, Amin R. Population size, density, and ranging behaviour in a key leopard population in the Western Cape, South Africa. Plos One. 2022;17(5):e0254507.

3. Amin R, Wilkinson A, Williams KS, Martins Q, Hayward J. Assessing the status of leopard in the Cape Fold Mountains using a Bayesian spatial capture-recapture model in Just Another Gibbs Sampler. Afr J Ecol. 2022;60(3):299-307.

4. Mann GK, O'Riain MJ, Parker DM. A leopard's favourite spots: Habitat preference and population density of leopards in a semi-arid biodiversity hotspot. J Arid Environ. 2020;181:104218.

5. Devens CH, Hayward MW, Tshabalala T, Dickman A, McManus JS, Smuts B, et al. Estimating leopard density across the highly modified human-dominated landscape of the Western Cape, South Africa. Oryx. 2021;55:34-45.

6. Skowno A, Raimondo D, Poole C, Fizziotti B, Slingsby J. South African national biodiversity assessment 2018 Volume 1: Terrestrial realm. South African National Biodiversity Institute. 2019.

7. South African Department: Forestry Fisheries & the Environment. SA National Land-Cover Dataset. Pretoria, South Africa; 2020.
